# Molecular Biomarkers Predict Pathological Complete Response of Neoadjuvant Chemotherapy in Breast Cancer Patients: Review

**DOI:** 10.3390/cancers13215477

**Published:** 2021-10-31

**Authors:** Ana Julia Aguiar de Freitas, Rhafaela Lima Causin, Muriele Bertagna Varuzza, Cassio Murilo Trovo Hidalgo Filho, Vinicius Duval da Silva, Cristiano de Pádua Souza, Márcia Maria Chiquitelli Marques

**Affiliations:** 1 Molecular Oncology Research Center, Barretos Cancer Hospital, Teaching and Research Institute, Barretos 14784-400, SP, Brazil; aaguiardefreitas@gmail.com (A.J.A.d.F.); rhafaela-lima@hotmail.com (R.L.C.); mbertagnav@gmail.com (M.B.V.); 2 Instituto do Câncer do Estado de São Paulo (ICESP), São Paulo 01246-000, SP, Brazil; cmtrovohidalgo@gmail.com; 3 Barretos Cancer Hospital, Barretos 14784-400, SP, Brazil; vinids@gmail.com (V.D.d.S.); crispadua10@gmail.com (C.d.P.S.); 4 Barretos School of Health Sciences, Dr. Paulo Prata–FACISB, Barretos 14785-002, SP, Brazil

**Keywords:** pathological complete response, neoadjuvant chemotherapy, breast cancer, molecular biomarkers

## Abstract

**Simple Summary:**

Breast cancer is the most common cancer in women worldwide. Although many studies have aimed to understand the genetic basis of breast cancer, leading to increasingly accurate diagnoses, only a few molecular biomarkers are used in clinical practice to predict response to therapy. Current studies aim to develop more personalized therapies to decrease the adverse effects of chemotherapy. Personalized medicine not only requires clinical, but also molecular characterization of tumors, which allows the use of more effective drugs for each patient. The aim of this study was to identify potential molecular biomarkers that can predict the response to therapy after neoadjuvant chemotherapy in patients with breast cancer. In this review, we summarize genomic, transcriptomic, and proteomic biomarkers that can help predict the response to therapy.

**Abstract:**

Neoadjuvant chemotherapy (NAC) is often used to treat locally advanced disease for tumor downstaging, thus improving the chances of breast-conserving surgery. From the NAC response, it is possible to obtain prognostic information as patients may reach a pathological complete response (pCR). Those who do might have significant advantages in terms of survival rates. Breast cancer (BC) is a heterogeneous disease that requires personalized treatment strategies. The development of targeted therapies depends on identifying biomarkers that can be used to assess treatment efficacy as well as the discovery of new and more accurate therapeutic agents. With the development of new “OMICS” technologies, i.e., genomics, transcriptomics, and proteomics, among others, the discovery of new biomarkers is increasingly being used in the context of clinical practice, bringing us closer to personalized management of BC treatment. The aim of this review is to compile the main biomarkers that predict pCR in BC after NAC.

## 1. Introduction

Breast cancer (BC) is the most commonly diagnosed malignancy and is responsible for the highest number of deaths among women worldwide [1]. Furthermore, BC is heterogeneous and presents different morphological and biological characteristics, thus leading to different clinical behaviors and responses [2]. Therefore, BCs are classified according to their characteristics, histological type, and expression of tumor markers, which develop from genetic and molecular changes in breast tissue cells [3,4].

Neoadjuvant chemotherapy (NAC) is an important treatment strategy for BC patients, with the aim of reducing staging and monitoring response to treatment for prognostic purposes, thereby increasing pathological complete response rate (pCR) [5]. pCR is an important long-term clinical outcome for patients with BC, as patients who achieve pCR with neoadjuvant therapy tend to have better disease-free survival (DFS) and overall survival (OS) compared with patients with residual invasive disease [6,7].

pCR is defined as the complete disappearance of all invasive breast carcinoma cells and axillary lymph nodes (ypT0/ypN0), and is determined pathologically in the resected tissue after NAC [8]. Predicting which patients will achieve pCR or have residual disease (RD) may help suggest and plan a specific treatment according to patient’s characteristics, thus enabling personalized therapy. Clinical staging, axillary lymph node status, and human epidermal growth factor receptor-2 (HER2) positivity are associated with cancer recurrence rates after NAC [9]. RD is defined by the presence of breast cancer cells in the tumor bed and/or positive lymph nodes after surgical removal. Patients with documented RD are usually associated with a worse prognosis than those who achieve pCR, although RD can have a heterogeneous prognosis in each patient [10,11]. Studies have attempted to identify molecular biomarkers that could monitor patients with early pCR and avoid overtreatment in this population. However, these markers require larger studies with long-term follow-up, and for this reason, they currently lack clinical validation [12,13].

The identification of efficient molecular markers that can predict sensitivity to chemotherapy, demonstrate higher rates of pCR, and identify patients that can benefit from NAC in clinical practice has been a challenge in many recent studies. However, molecular markers can be effective in avoiding unnecessary treatments and associated toxicities for BC patients that do not respond to NAC [14]. Since histologically similar tumors may demonstrate different prognoses and responses to therapy, some molecular subtypes of BC can have high rates of pCR to NAC, while others may not have the same benefits from being exposed to the same treatment. Therefore, there is a need for predictive biomarkers to select patients who will not benefit from NAC in order to offer new therapeutic approaches to these patients [15]. NAC offers an opportunity to identify biomarkers that are predictive of the response to such treatment in patients with BC.

**Figure 1:**
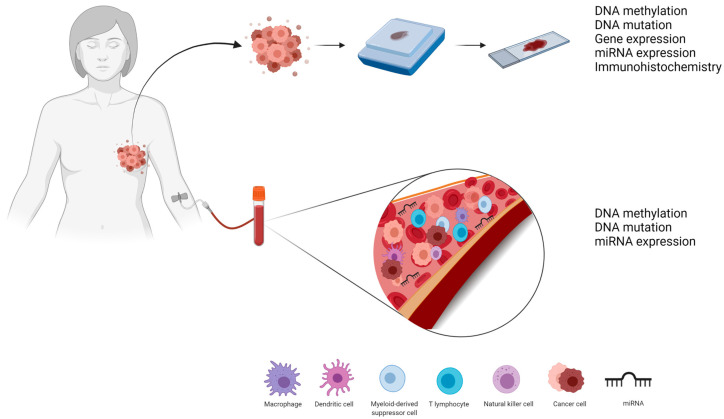
Tumor and liquid biopsies can help identify pCR biomarkers as they can provide information at the genomic (DNA methylation and DNA mutation), transcriptomic (mRNA and miRNA expression), and proteomic (immunohistochemistry) levels.

Biomarkers that use “omics” technologies, i.e., genomics, transcriptomics, and proteomics, in BC research have gained recognition in the scientific community. These omics analyses involve the identification of biomolecules responsible for each step of cell function control from DNA replication (genomics markers) to transcriptional events and post-transcriptional regulation (transcriptomic markers) to protein translation (proteomic markers). These markers can be identified not only in tumor tissues but also by liquid biopsy (Figure 1) [16,17], which could assist in the development of new drugs and in the identification and monitoring of patients who will respond and benefit from this treatment [18,19].

Despite the importance of pCR markers for therapy selection, we identified only a few studies that explored this potential and demonstrated that many molecules are differentially expressed at the genomic, transcriptomic, and proteomic levels, and can be used as effective biomarkers of NAC response.

## 2. Genomic: DNA as Biomarkers of NAC Response in BC Patients

The potential use of genomic markers for diagnosis and predicting prognosis, and response to treatment has been increasingly studied. DNA mutations, DNA methylation, and circulating tumor DNA (ctDNA) are among the main classes of genomic biomarkers. These molecules can be identified in tumor tissues or biofluids, such as blood, serum, or plasma samples.

### 2.1. DNA Mutation

Mutations in genes such as oncogenes or tumor suppressor genes have been widely studied because of their potential as predictors of prognosis. Furthermore, it is possible to predict their impact on tumor development and progression. Table 1 summarizes the mutations that predict pCR in patients with BC. Dysfunctions in DNA repair pathways can occur because of genetic mutations that compromise genomic integrity. Genomic instability is an important hallmark of carcinogenesis, and cellular machinery plays an important role in maintaining this stability [20]. For example, homologous recombination is necessary for repairing DNA double-strand breaks. Some genes have already been described in the literature, such as *BRCA1/2*, which encodes proteins necessary for homologous recombination repairing [13].

Several studies have evaluated many gene mutations to identify sensitive and specific biomarkers that can predict patient response to different treatments and assess their impact on development and tumor progression. In BC settings, germline mutations of *BRCA1/2* genes are frequent in patients with the triple negative breast cancer (TNBC) molecular subtype. A study evaluated the pCR rate in TNBC patients, with or without a mutation in one of these genes, who received NAC with epirubicin, cyclophosphamide, docetaxel, and bevacizumab. The findings showed that therapy with bevacizumab promoted pCR in patients with *BRCA1/2* mutations [21].

Another widely studied mutation is the *PIK3CA* gene mutation. This gene encodes the p110α catalytic subunit of the phosphatidylinositol 3-kinase signaling pathway, one of the intracellular pathways often related to BC [22]. Using whole exome sequencing (WES), Shi et al. identified a correlation between the *PIK3CA* gene mutation and resistance to trastuzumab treatment associated with non-pCR. Furthermore, these findings indicate that treatment with lapatinib provided better outcomes in patients with HER2-positive molecular phenotype BC who had *PIK3CA* driver mutation [23].

A second study also evaluated pCR based on the *PIK3CA* mutation in HER2-positive or TNBC patients who received NAC with paclitaxel and doxorubicin. Patients with TNBC also received bevacizumab and carboplatin. However, in this study, the researchers evaluated only exons 9 and 20, and not the complete gene, through DNA sequencing. By performing a multivariate analysis, it was possible to associate the *PIK3CA H1047R* hotspot mutation with non-pCR in TNBC patients [22].

A study evaluated pCR in patients with early BC who received NAC with capecitabine, docetaxel (for HER2-negative patients), and trastuzumab (HER2-positive patients). This analysis was performed by identifying mutations in the *TP53* gene, a tumor suppressor that is involved in the regulation of cell proliferation, survival, and genomic integrity in BC. The authors showed that the most frequent mutation was missense and that patients with the mutation had higher pCR rates, indicating the effectiveness of NAC [24].

In the context of identifying potential predictive genomic biomarkers, the expression of the *TOP2A* gene has been studied using the fluorescent in situ hybridization (FISH) technique. *TOP2A* encodes topoisomerase IIα, a key enzyme in DNA replication, and one of the molecular targets of anthracyclines, and is mutated in a significant percentage of HER2-overexpressing BC patients. Thus, several studies have evaluated the relationship between this gene and its ability to predict resistance to anthracyclines in BC. Desmedt et al. evaluated patients with estrogen receptor-negative (ER-negative) and HER2-positive molecular phenotypes who were treated with anthracycline (epirubicin) and taxane. The findings showed that *TOP2A* amplification was correlated with pCR in patients who received anthracycline alone [25].

Tibau et al. also evaluated the relationship between *TOP2A* and anthracycline resistance. Because this gene is located on chromosome 17, close to the centromere, the authors also evaluated the duplication of the centromere on this chromosome (*CEP17*), which may be a biomarker for genomic instability and DNA repair dysfunction. In this study, HER2-positive BC patients who underwent NAC with either fluorouracil, or taxanes combined with anthracyclines and cyclophosphamide, and were treated before trastuzumab approval, were evaluated. Multivariate analysis showed that the presence of *CEP17* duplication, as well as *TOP2A* amplification, showed a high percentage of pCR [26].

Besides point mutations, it is also possible to use biomarkers based on DNA expression profiles, such as the homologous recombination deficiency (HRD) score, which includes telomeric allelic imbalance (TAI, defined as the number of regions with allelic imbalance that extend to one of the subtelomeres), large-scale state transitions (LST, defined as the number of chromosomal breaks between adjacent regions), and loss of heterozygosity (LOH, defined as allele-specific copy number for each sub-chromosomal region). Kaklamani et al. investigated HRD associated with mutation status for *BRCA1/2* genes and *BRCA1* promoter methylation through DNA sequencing. An important finding was that patients who had a *BRCA1/2* germline mutation or methylation had an HRD score above the threshold. In this study, which aimed to evaluate these profiles based on DNA and protein expression as potential predictors of therapeutic response, it was possible to predict pCR to treatment with carboplatin and eribulin in patients with early stage TNBC [27].

### 2.2. DNA Methylation

DNA methylation, a type of epigenetic alteration that is involved in carcinogenesis, consists of the addition of a methyl group in the promoter region of a gene related to the tumor development process. These changes occur at high rates and contribute to the loss of epigenetic regulation, which can be crucial in early stages of carcinogenesis. The most common alteration is DNA hypermethylation of CpG dinucleotide islands, which increases the probability of sporadic mutation by deamination of 5-methylcytosine to thymine, resulting in point mutations and abnormal protein translation [28,29]. Some studies have shown that epigenetic mechanisms such as DNA methylation occur more often in patients who had pCR compared to women who had RD. Accordingly, Table 2 summarizes the studies that demonstrated whether DNA methylation could predict pCR in BC patients.

Almeida et al. investigated genome-wide DNA methylation patterns in BC patients and correlated the variations with gene expression data from The Cancer Genome Atlas (TCGA) and Molecular Taxonomy of Breast Cancer International Consortium (METABRIC) databases. Their study suggests that both hypermethylation and hypomethylation of CpG may be crucial events in BC and identifies three new diagnostic and prognostic biomarker candidates for DNA methylation [29].

Studies have demonstrated the consequences of DNA methylation in the cancer research landscape. Fujii et al. identified methylation of the promoter CpG island of the *HSD17B4* gene through genome-wide methylation analysis in tumor samples from patients with HER2-positive BC. Using DNA sequencing and performing a multivariate analysis, it was possible to predict pCR for treatment with trastuzumab, paclitaxel, or anthracycline through methylation of that gene that encodes the 17β-hydroxysteroid dehydrogenase type 4 enzyme [30].

Another study evaluated the response to NAC using DNA methylation profiles. Connolly et al. used a methylation panel, which investigated 10 genes (*HIST1H3C, AKR1B1, GPX7, HOXB4, TMEFF2, RASGRF2, COL6A2, ARHGEF7, TM6SF1,* and *RASSF1A*), to evaluate tumor tissue and serum samples from patients with HER2-negative BC. These genes were selected from previous studies [32,33] that identified their methylation in breast tumors at all stages and in the serum of patients with metastatic BC. By performing exploratory analyses with univariate and multivariate logistic regression models, it was possible to associate the high cumulative methylation index with non-pCR in patients who underwent NAC with carboplatin and nab-paclitaxel or vorinostat [31].

### 2.3. Circulating Tumor DNA

The detection of ctDNA through liquid biopsy has already been used in clinical practice to monitor cancer. However, studies have been performed to improve the technique and identify precise biomarkers. These molecules have been studied because they can be obtained using a minimally invasive approach as ctDNA is evaluated through plasma samples.

Malignant cells release cell-free DNA molecules into the bloodstream, thus allowing tumor progression. Studies have evaluated the potential of these molecules as prognostic factors and their response to treatment biomarkers. The first published study of ctDNA and BC was published more than a decade ago [34], and since then, several studies have examined techniques used to identify highly sensitive and specific biomarkers [35].

A recent study evaluated the efficacy of NAC with paclitaxel and/or anthracycline in BC patients using ctDNA expression through WES. This study identified that high expression of ctDNA was associated with non-pCR, thus suggesting it to be a considerable biomarker of early response prediction in the neoadjuvant setting in different molecular subtypes [36]. Moreover, this dynamic monitoring during treatment can facilitate the evaluation of new agents, providing greater sensitivity to the effectiveness of the treatment.

## 3. Transcriptomic: mRNA and miRNAs as Biomarkers of NAC Response in BC Patients

Studies have demonstrated that transcriptomic biomarkers can predict a patient’s response to NAC. The main classes of these biomarkers are the expression of genes and the miRNAs, which may originate from tumor or liquid biopsy.

### 3.1. Gene Expression Panels

With the discovery of microarray technology, it is possible to analyze the expression of several genes simultaneously. Thus, Perou et al. were able to use gene expression assays to identify five molecular subtypes in BC: (1) baseline as, Erb−B2+, (2) normal breast, (3) luminal A, (4) luminal B, and (5) luminal C [37]. Later, this classification underwent several modifications, and it was widely accepted as a method to identify the prognostic significance of BC, in which the ER+ HER2− and luminal A tumors demonstrated a better prognosis, while the baseline and non-baseline triple-negative tumors had worse prognosis [38].

Over the past few years and with the advancement of scientific research, it has been possible to identify several biomarkers for BC, and many gene expression signatures have become commercially available as prognostic tools for this neoplasm. Oncotype DX gene panels (RS; Genomic Health, Redwood City, CA, USA) [39], Mammaprint [40] (Agendia, Amsterdam, the Netherlands), EndoPredict (EP; Myriad Genetics, Cologne, Germany) [41], Prediction Analysis of Microarray 50 (PAM50) Risk of Recurrence, Prossigna Kit (Prosigna; NanoString Technologies, Seattle, WA, USA) [42], and breast cancer index (BCI; Biotheranostics, San Diego, CA, USA) [43] are some examples of panels that explore and derive conclusions about tumor recurrence and relapse [44,45]. The main multigene expression signatures (MES) used as biomarkers for pCR in BC that are currently available on the market are shown in Table 3.

Indeed, the biomarkers that predict patients’ response to NAC offer an opportunity for personalized service, better response rates to therapy, reduced adverse effects, and cost savings for the public health system by avoiding overtreatment in patients who will have non-pCR [45]. Because there are now different molecular signatures, some studies have pointed out that certain commercial gene expression panels may be useful in stratifying patients who will have pCR. Currently, the most commonly used panel is the Oncotype DX, which consists of a panel that assesses the expression of 21 genes in tumor tissue. The test result, considered as Recurrence Score, is able to provide information on the probability of tumor recurrence, as well as the chance of the patient presenting pCR in the face of NAC administration [46].

### 3.2. Differentially Expressed miRNA

From genome-wide miRNA expression analysis, it was possible to identify several miRNAs that were differentially expressed in BC tissue [47]. Since then, many studies have reported the importance of this molecule in different tumor phenotypes [48]. One recent approach was the ability of miRNA expression profiles to classify breast tumors according to histopathological variables, which are currently used to indicate responsiveness to neoadjuvant therapy [49,50,51,52,53,54,55]. As a result, these molecules are highlighted as potential predictive biomarkers that can allow the individualization of BC treatment and a better selection of patients who could respond to NAC.

Evidence has shown that miRNAs can be differentially expressed in the bloodstream of patients with pCR to NAC compared with patients with RD. Circulating miRNAs (ct-miRNAs) originate from the tumor tissue and migrate into the bloodstream, which makes it possible to identify the specific biological characteristics of the tumor [56,57]. With the advancement of technology in recent years, the detection of ct-miRNAs from body fluids has been made possible, and the evaluation of ct-miRNA expression has shown that it has great potential as a biomarker for early detection, drug resistance, tumor recurrence, and clinical outcome prediction of patients on cancer therapy [58], especially for monitoring of BC patient treatment [59].

Seven articles were identified in this context. These studies evaluated differential miRNA expression and investigated the association between miRNAs and pCR or non-pCR in BC patients who underwent NAC (Table 4). The results obtained from the high-throughput miRNA profile assessment identified four significant signatures between HER2-positive patient groups that received lapatinib at T0 and T1 and the group that received lapatinib and trastuzumab at T1, demonstrating promising evidence for future analyses using ct-miRNAs to assess the response to anti-HER2 agents. However, the authors stated that confirmatory studies in independent case series are needed to validate and evaluate the generalization of these ct-miRNA signatures. The data presented in this study may have direct implications for future clinical trials, as miRNA analyzed in plasma can be a promising strategy for predicting response to trastuzumab as monotherapy and can be used to guide de-escalation therapy [52]. Cosimo et al. identified increased levels of ct-miRNAs, from which ct-miR-148a-3p and ct-miR-374a-5p were significantly associated with pCR after NAC in patients with HER2-positive BC [49]. Using univariate and multivariate models, it was verified that miR-155 and miR-301 indicated a better pCR. This study evaluated the expression of miRNAs from the isolation of total plasma exosomes from patients with TNBC and HER2-positive patients before NAC. It was possible to identify a network of deregulated exosomal miRNAs with specific expression patterns in exosomes of HER2-positive and TNBC patients that are also associated with clinicopathological parameters and pCR within each molecular subtype of BC [51].

García-García et al. also demonstrated that miR-145-5p low expression was associated with high pCR rates in patients with TNBC who received cisplatin/doxorubicin-based neoadjuvant treatment. In contrast, patients with higher levels of miR-145-5p expression did not respond to chemotherapy regimens and had worse outcomes [53]. In addition, this study suggested that miR-145-5p could be a predictor of pCR. Our hypothesis is that patients with a worse prognosis may respond better because of the proliferative index. García-García et al. performed functional in vitro assays and verified that miR-145 mimics were able to decrease cell line proliferation of TNBC (MDA-MB-231), and a high expression level of miR-145-5p was identified in patients with non-pCR after NAC regimen [53]. On the other hand, another study demonstrated that the expression of ct-miR-21 could accurately distinguish clinical responders from non-responders, but it was not possible to distinguish those with pCR from those with RD [50].

Bearing in mind that ct-miRNAs, acting as potential predictive and prognostic biomarkers, may be able to identify patients who will have pCR allows us to individualize the treatment of BC and better select patients for NAC. Although rapid and continuous advances are being made in regard to the use of differentially expressed miRNAs as biomarkers of pCR prediction, this area of research still has many obstacles to overcome before its implementation in the management of BC patients’ clinical practice. To date, few studies have evaluated pCR after NAC treatment. Therefore, to validate these miRNA as effective biomarkers for the identification of patients who will achieve pCR, large clinical trials are needed to support these preliminary findings. Current obstacles to overcome include identifying methods for evaluating miRNA expression profiles that are specific, sensitive, and highly accurate at low cost. Additionally, research on the discovery of new biomarkers and more accessible technologies is essential, and the identification of a biomarker that could predict or potentially monitor the tumor’s response to NAC could revolutionize the way chemotherapeutic drugs are administered, bringing us closer to personalized management of BC.

## 4. Proteomic: Proteins as Biomarkers of NAC Response in BC Patients

Protein biomarkers are widely used in clinical practice to assess the prognosis of patients. Different studies have reported differential expression of proteins as biomarkers of pCR in molecular subtypes of BC (Table 5). Currently, many protein biomarkers have been identified in BC tissues and/or from the tumor-infiltrating immune system [60]. The advent of protein analysis in BC made it possible to obtain prognostic markers [61] and identify molecular subtypes [62] using immunohistochemistry (IHC). These biomarkers are currently available and can guide the clinical management of targeted therapy. IHC is a quick and inexpensive assay that provides important diagnostic and prognostic information [63,64].

Proliferation markers can predict systemic responses to NAC in some molecular subtypes of BC. Ki-67 is a non-histone nuclear protein expressed during all cell cycle phases, except the G0 phase. Therefore, Ki-67 is used as a marker for tumor proliferation [71]. This marker was identified by IHC analysis, where the levels of Ki-67 expression were associated with the percentage of tumor cells stained positively among the total number of malignant cells evaluated [72].

The use of Ki-67 has been reported in previous BC studies, which demonstrated that this protein expression can predict the response to NAC [73,74]. Yoshioka et al. demonstrated that a high Ki-67 expression in tumors before treatment was associated with higher rates of pCR, and a high Ki-67 expression in post-treatment tumors was strongly correlated with low DFS and OS, regardless of subtype [65].

Another protein that is related to pCR is carbonic anhydrase IX (CAIX), which is a transmembrane protein and one of the only two isoenzymes of carbonic anhydrase associated with tumors that may be involved in cell proliferation and transformation [66]. Alves et al. first described CAIX expression as a predictor of pCR and its association with DFS and OS in patients with locally advanced BC treated with NAC using doxorubicin, cyclophosphamide, and paclitaxel [66].

Studies have shown that in patients with TNBC, the immune system can influence the chemotherapy response. One example is programmed cell death-ligand 1 (PD-L1), a transmembrane protein expressed in a variety of cells, including epithelial cells, vascular endothelial cells, macrophages, myeloid dendritic cells, and B cells [75]. Cerbelli et al. investigated the role of PD-L1 expression in predicting the pathological response to NAC in TNBC. Before NAC, biopsies showed that PD-L1 in ≥25% of tumor cells predicted pCR in TNBC. A possible explanation for these findings is that PD-L1 expression may be associated with a subpopulation of TNBC with more aggressive behavior, with a probability of responding to chemotherapy [67].

Similarly, FK506 binding protein 12 (FKBP12) is a cytoplasmic protein expressed with multiple functions in the transduction of cell signaling [76] and has been reported as a predictive biomarker for the effectiveness of anthracycline-based chemotherapy in BC. Xing et al. demonstrated that the loss of FKBP12 was specifically correlated with poor prognosis and increased resistance to anthracycline-based chemotherapy. Patients with low FKBP12 expression had a significantly lower rate of pCR [68].

A study with a female Japanese population with locally advanced BC showed that lower levels of MGMT protein expression were associated with higher pCR rates when compared with women with normal expression levels of MGMT protein [69]. MGMT is a DNA repair protein that removes alkylating agents from DNA [77].

Furthermore, annexins are a large multifunctional family of phospholipid-binding proteins regulated by Ca^2+^ [78]. Annexin A1 (ANXA1) is linked to phospholipids involved in inflammation, immune response, and reactivity of mast cells and is associated with the aggressive phenotype of TNBC [79]. Annexin A2 (ANXA2) is a calcium-binding cytoskeleton protein located on the extracellular surface of endothelial cells and in various types of tumor cells [80]. It has been shown that the expression of ANXA2 in breast tumors can be a biomarker for predicting BC outcome in high-risk groups [81]. Chuthapisith reported that the proteins ANXA1 and ANXA2 are predictors of pCR, as it was demonstrated that the presence of ANXA2 in conjunction with ANXA1 could be a potential marker of non-pCR in BC [70].

## 5. Final Considerations

Our review found several studies that evaluated potential molecular markers as predictors of pCR. The main markers are gene mutations, DNA methylation, and the expression of miRNAs and proteins. Reaching ypT0/ypN0 is strongly associated with a great impact on improving overall and progression-free survival in BC patients, as it is independent of nodal status, and apparently of greater benefit in patients with TNBC [8,82]. Predicting which patients will benefit from NAC is one of the main reasons for researching non-invasive response markers. Despite the existence of various studies to identify biomarkers associated with pathological response, there is still no ideal molecular marker that can be used in clinical practice to distinguish resistant and sensitive patients and, thus, help define possible changes in treatment for patients without pCR [83]. The aim of this review was to compile the main genomic, transcriptomic, and proteomic signatures that were tested for pCR.

The search for biomarkers has been the target of many studies, as they can be used for diagnosis, prognosis, and drug selection in BC [84,85]. There are biomarkers that have already been validated by clinical trials and, therefore, may be available to assist in clinical practice, and promising biomarkers that still need to be better explored and validated [83].

The evolution of methods, such as artificial intelligence-powered imaging analysis, use of high-performance molecular profiling, and computational tools allow the implementation of personalized medicine and aid in prognosis and risk stratification. These methods can also be used for scaling or avoiding therapies, and predicting response to treatment [86,87].

The identification of clinically useful biomarkers is challenging due to several limitations, including tumor heterogeneity, since a single biomarker may not have sufficient sensitivity and specificity to predict response to therapy and tumor behavior [88]. The lack of standardized protocols and precise cutoff values, the need for a complete assessment of sensitivity, specificity and reproducibility are also obstacles for the validation of these biomarkers in clinical practice [89]. The development of affordable biomarkers also poses a challenge due to the high cost needed for use in clinical practice [90]. Therefore, defining which biomarkers that might be clinically applicable to discern between responders and non-responders to NAC is a challenge.

**Figure 2:**
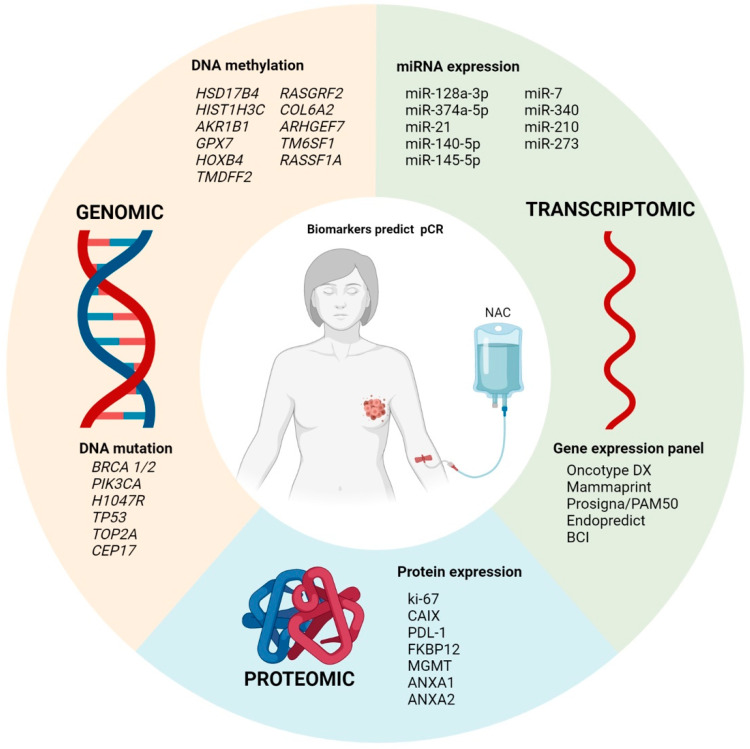
Summary of the main pCR markers at genomic, transcriptomic, and proteomic levels.

In this review, we highlighted several studies that used the omics approach to identify new biomarkers as potential predictors for target therapy (Figure 2). The use of these biomarkers, although scarcely used in clinical practice, has been shown to be sufficiently accurate to distinguish patients who will achieve pCR. However, further studies with larger cohorts and clinically controlled and randomized groups need to be conducted to validate these findings.

Current research has not yet identified any predictive molecular biomarkers for pCR in BC patients, which are sufficiently robust and can be used in the clinical management of patients with pCR or RD. Hence, it is essential to identify genomic, transcriptomic, and proteomic markers that are specific, sensitive, and accurate. In this review, we demonstrated that different biomarkers may be important in predicting a patient’s response to distinct treatments. Thus, it can minimize the adverse effects and toxicity commonly caused by these drugs and anticipate cases in which patients will not benefit from certain drugs.

## Figures and Tables

**Table 1:** DNA mutations as biomarkers of pCR or non-pCR after NAC in BC patients.

Author, Year	Specimens	DNA Mutation Biomarkers	NAC	IHC Subtypes (*n*)	Outcome	Ref.
Fasching et al., 2018	Plasma	*BRCA1/2*	Epirubicin Cyclophosphamide Docetaxel Bevacizumab	TNBC (*n* = 493)	pCR	[21]
Guo et al., 2020	FFPE	*PIK3CA* *H1047R*	Paclitaxel Doxorubicin Bevacizumab Carboplatin	TNBC (*n* = 92)	non-pCR	[22]
Shi et al., 2017	Frozen tissue	*PIK3CA*	Lapatinibe Trastuzumab	HER2+ (*n* = 207)	non-pCR	[23]
Gluck et al., 2011	Frozen tissue	*TP53*	Capecitabine Docetaxel Trastuzumab	HER2− (*n* = 99) HER2+ (*n* = 38)	pCR	[24]
Desmedt et al., 2011	FFPE Frozen tissue	*TOP2A*	Anthracycline Epirubicin Taxanes	ER− HER2+ (*n* = 106)	pCR	[25]
Tibau et al., 2014	FFPE	*TOP2A* *CEP17*	Fluorouracil Epirubicin Cyclophosphamide Doxorubicin Docetaxel	Non-classification (*n* = 140)	pCR	[26]

pCR, pathological complete response; non-pCR, non-pathological complete response; NAC, neoadjuvant chemotherapy; IHC subtypes, molecular subtypes identified by immunohistochemistry; HER2+, human epidermal growth factor receptor-2 positive; HER2−, human epidermal growth factor receptor-2 negative; TNBC, triple-negative breast cancer; ER− HER2+: estrogen receptor-negative and human epidermal growth factor receptor-2 positive; FFPE: formalin-fixed, paraffin-embedded.

**Table 2:** DNA methylation as a biomarker of pCR or non-pCR after NAC in BC patients.

Author, Year	Specimens	DNA Methylation Biomarkers	NAC	IHC Subtypes	Outcome	Ref.
Fujii et al., 2017	FFPE	*HSD17B4*	Trastuzumab Paclitaxel Anthracycline	HER2+ (*n* = 67)	pCR	[30]
Connolly et al., 2018	FFPE Serum	*HIST1H3C* *AKR1B1* *GPX7* *HOXB4* *TMEFF2* *RASGRF2* *COL6A2* *ARHGEF7* *TM6SF1* *RASSF1A*	Carboplatin Nab-paclitaxel Vorinostat	HER2− (*n* = 61)	non-pCR	[31]

pCR, pathological complete response; non-pCR, non-pathological complete response; NAC, neoadjuvant chemotherapy; IHC subtypes, molecular subtypes identified by immunohistochemistry; HER2+, human epidermal growth factor receptor-2 positive; HER2, human epidermal growth factor receptor-2 negative; FFPE, formalin-fixed, paraffin-embedded.

**Table 3:** Commercial panels for prognostic evaluation of BC patients using mRNA gene expression.

Panel	Techonology	Genes
Oncotype DX	RT-qPCR	*ACTB; BAG1; BCL2; BIRC5; CCNB1; CD68; CTSL2; ESR1; GAPDH; GRB7; GSTM1; GUS; HER2; Ki-67; MMP11; MYBL2; PGR; RPLPO; SCUBE2; STK15; TRFC*
Mammaprint	NGS	*AA555029_RC; ALDH4A1; AP2B1; AYTL2; BBC3; C16orf61; C20orf46; C9orf30; CCNE2; CDC42BPA; CDCA7; CENPA; COL4A2; DCK; DIAPH3; DTL; EBF4; ECT2; EGLN1; ESM1; EXT1; FGF18; FLT1; GMPS; GNAZ; GPR126; GPR180; GSTM3; HRASLS; IGFBP5; JHDM1D; KNTC2; LGP2; LIN9; LOC100131053; LOC100288906; LOC730018; MCM6; MELK; MMP9; MS4A7; MTDH; NMU; NUSAP1; ORC6L; OXCT1; PALM2; PECI; PITRM1; PRC1; QSCN6L1; RAB6B; RASSF7; RECQL5; RFC4; RTN4RL1; RUNDC1; SCUBE2; SERF1A; SLC2A3; STK32B; TGFB3; TSPYL5; UCHL5; WISP1; ZNF533*
Prosigna/ PAM50	Nanostring	*ACTR3B; ANLN; BAG1; BCL2; BIRC5; BLVRA; CCNB1; CCNE1; CDC20; CDC6; CDCA1; CDH3; CENPF; CEP55; CXXC5; EGFR; ERBB2; ESR1; EXO1; FGFR4; FOXA1; FOXC1; GPR160; GRB7; KIF2C; KNTC2; KRT14; KRT17; KRT5; MAPT; MDM2; MELK; MIA; MKI-67; MLPH; MMP11; MYBL2; MYC; NAT1; ORC6L; PGR; PHGDH; PTTG1; RRM2; SFRP1; SLC39A6; TMEM45B; TYMS; UBE2C; UBE2T*
EndoPredict	RT-qPCR	*AZGP1; BIRC5; CALM2; DHCR7; HBB; IL6ST; MGP; OAZ1; RBBP8; RPL37A; STC2; UBE2C*
BCI	RT-qPCR	*BUB1B; CENPA; HOXB13; IL17BR; NEK2; RACGAP1; RRM2*

RT-qPCR, reverse transcriptase quantitative polymerase chain reaction; NGS, next-generation sequencing; PAM50, prediction analysis of microarray 50; BCI, breast cancer index.

**Table 4:** miRNAs as biomarkers of pCR or non-pCR after NAC in BC patients.

Author, Year	Specimens	miRNA Biomarkers	NAC	IHC Subtypes (*n*)	Outcome	Ref.
Cosimo et al., 2020	Plasma	ct-miR-148a-3p	Lapatinib Trastuzumab Paclitaxel	HER2+ (*n* = 52)	pCR	[49]
		ct-miR-374a-5p				
Liu et al., 2019	Serum	ct-miR-21	Taxotere Paraplatin Trastuzumab	HER2+ (*n* = 83)	non-pCR	[50]
Stevic et al., 2018	Plasma (exosomes)	18 exosomal miRNAs	Paclitaxel Doxorubicin Carboplatin	HER2+ (*n* = 211) TNBC (*n* = 224)	pCR	[51]
Cosimo et al., 2019	Plasma	ct-miR-140-5p	Lapatinib Trastuzumab Paclitaxel	HER2+ (*n* = 429)	non-pCR	[52]
García-García et al., 2019	FFPE	miR-145-5p	Cisplatin Doxorubicin	TNBC (*n* = 32)	pCR	[53]
Raychaudhuri et al., 2017	FFPE	miR-7	Epirrubicin Paclitaxel Cyclophosphamide Docetaxel	ER+ (*n* = 41) PR+ (*n* = 37) HER2+ (*n* = 36)	pCR	[54]
		miR-340				
Müller et al., 2014	Serum	ct-miR-21	Lapatinib Trastuzumab	HR+ (*n* = 71) HER2+ (*n* = 127)	non-pCR	[55]
		ct-miR-210				
		ct-miR-373				

pCR: pathological complete response; non-pCR: non-pathological complete response; NAC: neoadjuvant chemotherapy; IHC subtypes: molecular subtypes identified by immunohistochemistry; HER2+: human epidermal growth factor receptor-2 positive; TNBC: triple-negative breast cancer; ER+: estrogen receptor positive; PR+: progesterone receptor positive; HR+: hormone receptor positive; FFPE: formalin-fixed, paraffin embedded.

**Table 5:** Proteins as biomarkers of pCR or non-pCR after NAC in BC patients.

Author, Year	Specimens	Protein Biomarkers	NAC	IHC Subtypes (*n*)	Outcome	Ref.
Yoshioka et al., 2015	FFPE	Ki-67	Anthracycline Taxane-based	Luminal A (*n* = 8) Luminal B (*n* = 22) ER+ HER2+ (*n* = 11) ER− HER2+ (*n* = 12) TNBC (*n* = 11)	pCR	[65]
Alves et al., 2019	FFPE	CAIX	Doxorubicin Cyclophosphamide Paclitaxel	Luminal A (*n* = 22) Luminal B (*n* = 77) Luminal B HER2+ (*n* = 46) HER2 (*n* = 20) TNBC (*n* = 31)	pCR	[66]
Cerbelli et al., 2017	FFPE	PDL-1	Doxorubicin Cyclophosphamide Paclitaxel	TNBC (*n* = 54)	pCR	[67]
Xing et al., 2019	FFPE	FKBP12	5-florouracil Epirubicin Cyclophosphamide	Luminal HER2− (*n* = 334) HER2+ (*n* = 102) TNBC (*n* = 88)	pCR	[68]
Nakai et al., 2012	FFPE	MGMT	Anthracycline Taxane	TNBC (*n* = 32)	pCR	[69]
Chuthapisith et al., 2009	FFPE	ANXA1	Adriamycin Cyclophosphamid Docetaxel	Non-classification (*n* = 40)	non-pCR	[70]
		ANXA2				

pCR: pathological complete response; non-pCR: non-pathological complete response; NAC: neoadjuvant chemotherapy; IHC subtypes: molecular subtypes identified by immunohistochemistry; HER2+: human epidermal growth factor receptor-2 positive; HER2−: human epidermal growth factor receptor-2 negative; ER+ HER2+: hormone receptor positive and human epidermal growth factor receptor-2 positive; ER− HER2+: hormone receptor negative and human epidermal growth factor receptor-2 positive; TNBC: triple-negative breast cancer; FFPE: formalin-fixed, paraffin-embedded.
